# Intermarriage and mortality among Finnish migrants in Sweden: a prospective register study using binational data

**DOI:** 10.1093/eurpub/ckae179

**Published:** 2024-11-20

**Authors:** Kaarina Korhonen, Agneta Cederström, Pekka Martikainen, Olof Östergren

**Affiliations:** Helsinki Institute for Demography and Population Health, University of Helsinki, Helsinki, Finland; Max Planck–University of Helsinki Center for Social Inequalities in Population Health, University of Helsinki, Helsinki, Finland; Department of Public Health Sciences, Stockholm University, Stockholm, Sweden; Helsinki Institute for Demography and Population Health, University of Helsinki, Helsinki, Finland; Max Planck–University of Helsinki Center for Social Inequalities in Population Health, University of Helsinki, Helsinki, Finland; Max Planck Institute for Demographic Research, Rostock, Germany; Department of Public Health Sciences, Stockholm University, Stockholm, Sweden; Aging Research Center, Karolinska Institutet, Stockholm, Sweden

## Abstract

Conjugal ties may contribute to a convergence of health behaviours between migrants and natives, but the association between intermarriage and health outcomes remains understudied. We investigated mortality patterns among Finnish migrants in Sweden according to the spouse’s country of birth and compared these patterns with those observed in the native populations of both Sweden and Finland. Leveraging register data from Sweden and Finland, we identified all married Finnish migrants aged 40–64 and their spouses in Sweden in 1999 and corresponding reference groups in both countries. We used a combination of direct matching and inverse probability weighting to adjust for sociodemographic differences between the groups. We followed individuals for all-cause, alcohol-related, smoking-related, and cardiovascular disease (CVD) mortality during 2000–17. Accounting for sociodemographic characteristics, Finnish migrant men married to Swedish-born as opposed to Finnish-born spouses showed lower all-cause [incidence rate ratio (IRR) 0.94, 95% confidence interval (CI) 0.90–0.98], and CVD mortality (IRR 0.88, 95% CI 0.81–0.95), levels more akin to native Swedes. Migrant women with Swedish-born spouses instead had higher smoking-related mortality (IRR 1.41, 95% CI 1.24–1.61) than those married to Finnish-born spouses, mirroring the higher smoking-related mortality of native Swedish women. Individual-level regression analysis on migrants further indicated lower alcohol-related mortality for intermarried men, adjusted for duration of marriage (IRR 0.74, 95% CI 0.56–0.98). These findings suggest that intermarriage with a native spouse can facilitate the convergence of health behaviours and behaviour-related mortality between migrants and natives.

## Introduction

Amidst the expanding scope of international migration, the health of migrant populations has emerged as a crucial public health issue within many industrialized societies [1]. With migrants increasingly contributing to the composition of these societies, understanding the distinct health needs and challenges they encounter becomes imperative. However, migrants constitute a dynamic population moving across diverse contexts, which introduces various theoretical and methodological complexities into the analysis [2]. Therefore, analysing the intricate processes that underlie migrant health presents a challenging endeavour.

Several factors contribute to the health status of migrants, encompassing conditions in their country of origin, circumstances surrounding the migration event, as well as conditions in the destination country [3]. As migrants interact with the new environment, their living conditions, social relations, and behavioural patterns change, potentially leading to a convergence in health status towards that of the native population [4, 5].

Intermarriage with natives is often considered marker of advanced integration [6, 7]. Marrying a native is likely more common for migrants who have more extensive social connections with the native community and for those who have adopted prevailing social norms and language. Intermarriage in itself may also encourage a change in values and behaviours since conjugal ties may involve sustained close contact, shared decision-making, mutual support, and responsibility over each other’s well-being [8, 9]. Marriage between migrants and natives may thus facilitate the convergence of health behaviours and health status.

The existing body of literature has explored intermarriage between migrants and natives in relation to several processes, such as labour market performance and earnings [10], child outcomes [11–13], and mental health [14–16]. However, there remains a considerable gap in understanding how intermarriage can affect the somatic health of migrants. While intermarriage effects have been studied concerning specific somatic health outcomes like suicide [17] and COVID-19 mortality [18], broader investigations into the overall physical health and health risk behaviour outcomes associated with intermarriage are notably limited.

This study aims to address this gap by analysing whether the spouse’s country of birth is related to overall and cause-specific mortality among Finnish migrants in Sweden. This migrant population provides a unique opportunity for analysis. Migration between Finland and Sweden predominately took place in the 1960s and 1970s, and Finnish migrants in Sweden are now at an ideal age for studying mortality patterns. Furthermore, leveraging register data from both countries enables a comprehensive comparison of migrants with native populations in both the origin and destination countries accounting for sociodemographic characteristics [19], as well as marital and childbearing histories.

Contrary to the commonly observed migrant mortality advantage [20, 21], Finnish migrants in Sweden experience a mortality disadvantage relative to the native Swedish population, particularly among men [22–24]. This disadvantage is largely associated with lower socioeconomic position and attributable to higher rates of alcohol- and smoking-related mortality and cardiovascular disease (CVD) mortality [22]. Past research also suggests that mortality from these causes among Finnish migrants falls between those of the native populations of Finland and Sweden, and the disadvantage relative to the native Swedes tends to diminish over time spent in Sweden, especially among men [19].

We examine the impact of intermarriage on all-cause mortality and mortality related to health risk behaviours, i.e. mortality from alcohol, smoking, and CVD. Recognizing that health behaviours are contingent upon specific socioeconomic and demographic processes before and after migration, we leverage longitudinal population data from both Finland and Sweden which allows us to compare migrant groups with corresponding sociodemographic segments in both countries, as well as identify the spouse’s country of birth.

## Methods

We used linked administrative registers from Sweden and Finland, encompassing comprehensive data on sociodemographic information including age, sex, country of birth, migration events, income, educational attainment, marital status, and causes of death. These registers include links to spouses, allowing for the identification of the spouse’s country of birth. We identified all married Finnish-born individuals residing in Sweden and living with their spouses in 1999. We restricted the age range to 40–64 years (in 1999)—a period typically associated with active engagement in the labour market and concluded family formation. We then divided the sample into (1) Finnish-born migrant men (*n* = 13 867) and women (*n* = 14 803) who were married to a Finnish-born individual and (2) Finnish migrant men (*n* = 8350) and women (*n* = 16 138) who were married to a Swedish-born individual. We omitted migrants who were married to individuals with any other country of birth (*n* = 3040). We also identified (3) Swedish-born men (*n* = 685 855) and women (*n* = 702 217) who resided in Sweden and were married to a native Swede and (4) Finnish-born men (*n* = 511 837) and women (*n* = 513 438) who resided in Finland and were married to a native Finn, using the same age- and period restrictions. We excluded individuals who did not co-reside with their spouses.

We obtained information on education in 1999 (compulsory, intermediate, or tertiary) and income in 1994–99. Income was defined as the 6-year average household disposable income, adjusted for household composition by dividing income by the square root of the total household size and divided into quintiles for all four groups. For the migrants, we additionally obtained information indicating if they had at least one child registered in Sweden between 1980 and 2016, as well as the presence of at least one child residing in the household in 1999, using the total population register. We also collected information on the years of marriage and migration to determine whether the couple had married before or after migrating to Sweden. The study was approved by the Statistics Finland Board of Ethics (permit no. TK/3343/07.03.00/2023) and the Central Ethics Review Board of Sweden (Dnr Ö 25-2017).

We followed all individuals for mortality from 2000 through 2017, right censoring at emigration. Given the long follow-up, we treated age as a time-varying covariate, allocating deaths to the age at the time of occurrence rather than age at baseline. Besides deaths from any cause, we identified deaths related to alcohol and smoking and deaths from CVD using the 10th revision of the International Classification of Diseases (ICD-10) codes in the cause of death registers of Sweden and Finland. Alcohol-related deaths were identified by the following underlying or contributing causes: Alcohol-related disorders (F10.09), degeneration of nervous system due to alcohol (G31.2), alcoholic cardiomyopathy (I42.6), alcoholic gastritis (K29.2), alcoholic liver disease (K70), alcohol-induced acute pancreatitis (K85.2), and alcohol-induced chronic pancreatitis (K86.0). Smoking-related deaths were identified as malignant neoplasms of the respiratory and intrathoracic organs (ICD-10 codes C30–39) or chronic lower respiratory diseases (J40–47) as underlying causes. CVD deaths were identified as hypertensive diseases (I10-15), ischaemic heart disease (I20-25), vascular syndromes of the brain in cerebrovascular diseases (G45), cerebrovascular diseases (I60-69 and G46), or diseases of arteries, arterioles, and capillaries (I70-79) as underlying causes.

### Analytic strategy

The analytic strategy of the study consists of two parts. First, we analysed binational data to compare mortality patterns between migrants and the native populations of both countries. Second, we restricted the sample to Finnish migrants married to either Finnish-born or Swedish-born spouses and analysed individual-level data from Sweden to provide a more comprehensive understanding of the dynamics between intermarriage and mortality.

The first part of the analysis compares mortality patterns between Finnish migrants with Swedish-born spouses, Finnish migrants with Finnish-born spouses, native Swedes residing in Sweden with Swedish-born spouses, and native Finns residing in Finland with a Finnish-born spouse. Because the merging of individual-level data across country borders is prohibited due to data protection regulations [25], we used a combination of direct matching and inverse probability weighting to adjust for differences in age, sex, education, and income between the four groups without sharing individual-level data across borders.

Direct matching was employed to adjust for sociodemographic differences between the migrants and native-born populations in Sweden and Finland. Each migrant was paired with two randomly selected married controls in Finland and two in Sweden, matching their exact combination of age, sex, education, and income. Instead of sharing individual-level data, we merged aggregated numbers of person-years and deaths obtained from the Finnish dataset with the Swedish data.

In the next step, we used inverse probability weighting to adjust for sociodemographic differences based on the spouse’s country of birth within the Finnish migrant sample. Given the similar group size of Finnish migrants married to Finnish-born and Swedish-born spouses, direct matching was not a viable solution due to insufficient number of potential controls. We thus calculated the propensity of belonging in the treatment group (i.e. being married to a native Swede) based on sex-stratified logistic regression models where the spouse’s country of birth was the dependent variable and education and income, interacted with age, were the independent variables. After identifying the controls and applying the weights, the distribution of age, income, and education assumed the average distribution of all married migrants, allowing for direct comparison of mortality rates between the four groups (Table 1).

**Table 1.** The observed and matching/weighting adjusted distributions of native Swedes, Finnish migrants in Sweden with Swedish-born and Finnish-born spouses, and native Finns in Finland aged 40–64 years and married in 1999, and number of person-years and all-cause deaths in 2000–17

		Native Swedes	Migrants, Swedish-born spouse	Migrants, Finnish-born spouse	Native Finns in Finland	Native Swedes	Migrants, Swedish-born spouse	Migrants, Finnish spouse	Native Finns in Finland
		Observed	Observed	Observed	Observed	Matched	Weighted	Weighted	Matched
Men	Income (%)								
	Lowest quintile	5.3	7.1	8.0	10.0	7.7	7.9	7.7	7.7
	2nd	13.0	15.5	18.4	18.3	17.3	17.8	17.5	17.3
	3rd	23.0	24.9	27.9	22.2	26.7	26.7	26.7	26.7
	4th	28.2	29.5	29.0	24.1	29.8	29.4	29.7	29.8
	Highest quintile	30.5	23.1	15.7	25.4	18.5	18.3	18.4	18.5
	Education (%)								
	Compulsory	28.4	33.7	52.9	35.4	45.7	45.8	45.7	45.7
	Intermediate	48.3	50.0	40.6	33.7	44.1	44.0	44.1	44.1
	Tertiary	23.4	16.2	6.5	30.9	10.2	10.2	10.2	10.2
	Person-years	12 152 015	156 387	228 448	8 579 139	752 015	156 387	228 448	732 775
	*N*	685 855	8350	13 867	511 837	44 434	8350	13 867	44 434
	Deaths	87 736	1533	2837	83 303	6356	1533	2837	8956
Women	Income (%)								
	Lowest quintile	6.2	6.9	8.7	10.2	7.7	7.9	7.8	7.7
	2nd	14.6	15.1	20.0	18.1	17.5	17.6	17.6	17.5
	3rd	23.0	21.9	28.0	21.8	24.8	24.9	24.9	24.8
	4th	26.9	27.1	28.5	24.1	27.7	27.5	27.5	27.7
	Highest quintile	29.3	29.1	14.8	25.7	22.3	22.1	22.1	22.3
	Education (%)								
	Compulsory	24.6	24.6	50.0	35.6	36.8	36.9	36.8	36.8
	Intermediate	47.0	46.8	38.9	35.4	43.0	43.0	43.0	43.0
	Tertiary	28.4	28.6	11.0	29.0	20.2	20.2	20.2	20.2
	Person-years	12 466 657	305 928	253 642	8 903 568	1 066 657	305 928	253 642	1 069 678
	*N*	702 217	16 138	14 803	513 438	61 882	16 138	14 803	61 882
	Deaths	65 767	1857	1815	45 887	6278	1857	1815	6161

We calculated incidence rate ratios (IRR) for all-cause, alcohol-related, smoking-related, and CVD mortality using Poisson regression. We show results for sex-stratified models comparing the weighted sample of married migrants and matched controls in Sweden and Finland. Age-adjusted IRRs, that do not account for education and income, are provided in Supplementary Fig. S1. While our primary focus is married migrants and natives, Supplementary Fig. S2 presents this exercise repeated in the nonmarried population.

In the second part, by restricting the analysis to Finnish-born migrants residing in Sweden, we were able to consider migrant-specific characteristics and analyse regression models on individual-level data. We fit a series of Poisson regressions and expanded the analyses to include adjustments for the presence of children in Sweden and the household, and the duration of marriage (divided into tertiles). In a final step, we further categorized migrants based on marriage status at the time of migration, i.e. whether the marriage was formed before or after migrating to Sweden. All analyses are adjusted for age, education, and income and are performed for men and women separately.

## Results

In 1999, Sweden was home to 22 217 married Finnish migrant men and 30 941 married Finnish migrant women between the ages of 40 and 64. Among them, 8350 (37.6%) men and 16 138 (52.2%) women were married to a native Swede. Table 1 presents the observed and matching/weighting adjusted distributions of sociodemographic characteristics of the study population. Migrants married to Swedish-born spouses had higher education and higher household incomes compared to migrants married to Finnish-born spouses.

**Figure 1.**
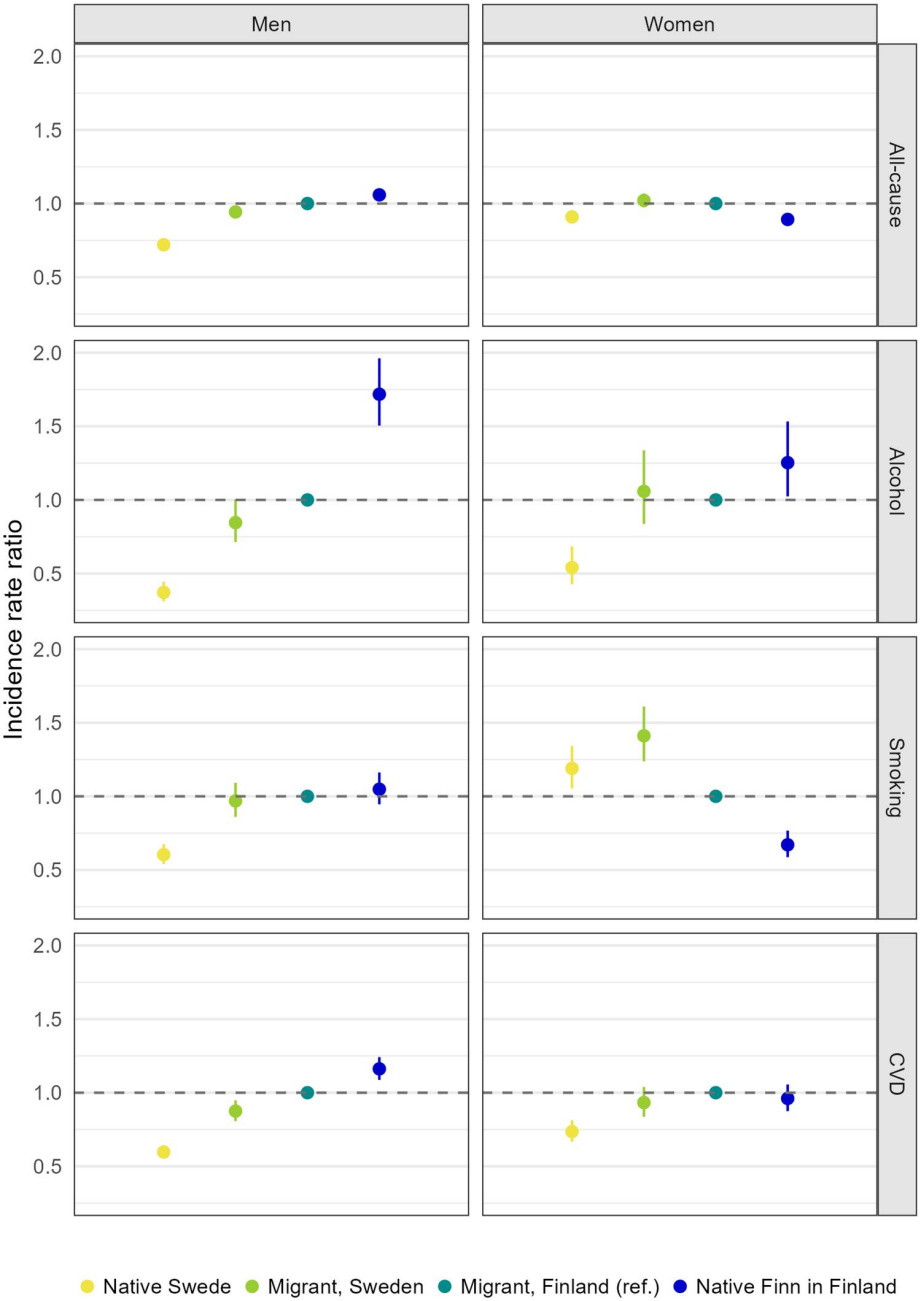
IRRs for all-cause and cause-specific mortality among Finnish migrants in Sweden by spouse’s country of birth as well as married native Swedes and Finns in 2000–17. IRRs adjusted for age, income, and educational attainment.

In Fig. 1, IRRs for all-cause and cause-specific mortality are shown for Finnish migrants in Sweden categorized by the spouse’s country of birth, as well as for native Swedes and native Finns in Finland. The IRRs are adjusted for age, income, and education through weighting and matching. Finnish migrant men who were married to Swedish-born spouses had lower all-cause (IRR = 0.94, 95% CI 0.90–0.98) and CVD mortality (IRR = 0.88, 95% CI 0.81–0.95) compared to migrant men married to Finnish-born spouses. Consequently, the mortality rates from these causes among intermarried migrant men resembled more closely those of native Swedes (IRR = 0.72, 95% CI 0.69–0.75 for all-cause mortality; IRR = 0.60, 95% CI 0.55–0.64 for CVD mortality), while mortality of migrant men married to a fellow Finn were closer to those of native Finns living in Finland (IRR = 1.06, 95% CI 1.02–1.10 for all-cause mortality; IRR = 1.16, 95% CI 1.09–1.24 for CVD mortality). A similar tendency was observed for alcohol-related mortality, but the difference was not statistically significant. Intermarriage was not associated with smoking-related mortality among men.

Among migrant women, having a Swedish-born spouse, in contrast to a Finnish-born spouse, was instead related to increased mortality from smoking-related causes (IRR = 1.41, 95% CI 1.24–1.61). The level of smoking-related mortality of intermarried migrant women was closer to that observed among native Swedish women (IRR = 1.19, 95% CI 1.05–1.34) and those married to Finnish-born spouses exhibited a level closer to the one observed among native Finns in Finland (IRR = 0.67, 95% CI 0.59–0.77). Although the difference was not statistically significant, CVD mortality among intermarried migrant women also resembled more the (lower) level of CVD mortality among native Swedes than the native Finns in Finland. Intermarriage was not associated with all-cause and alcohol-related mortality among women.

**Figure 2.**
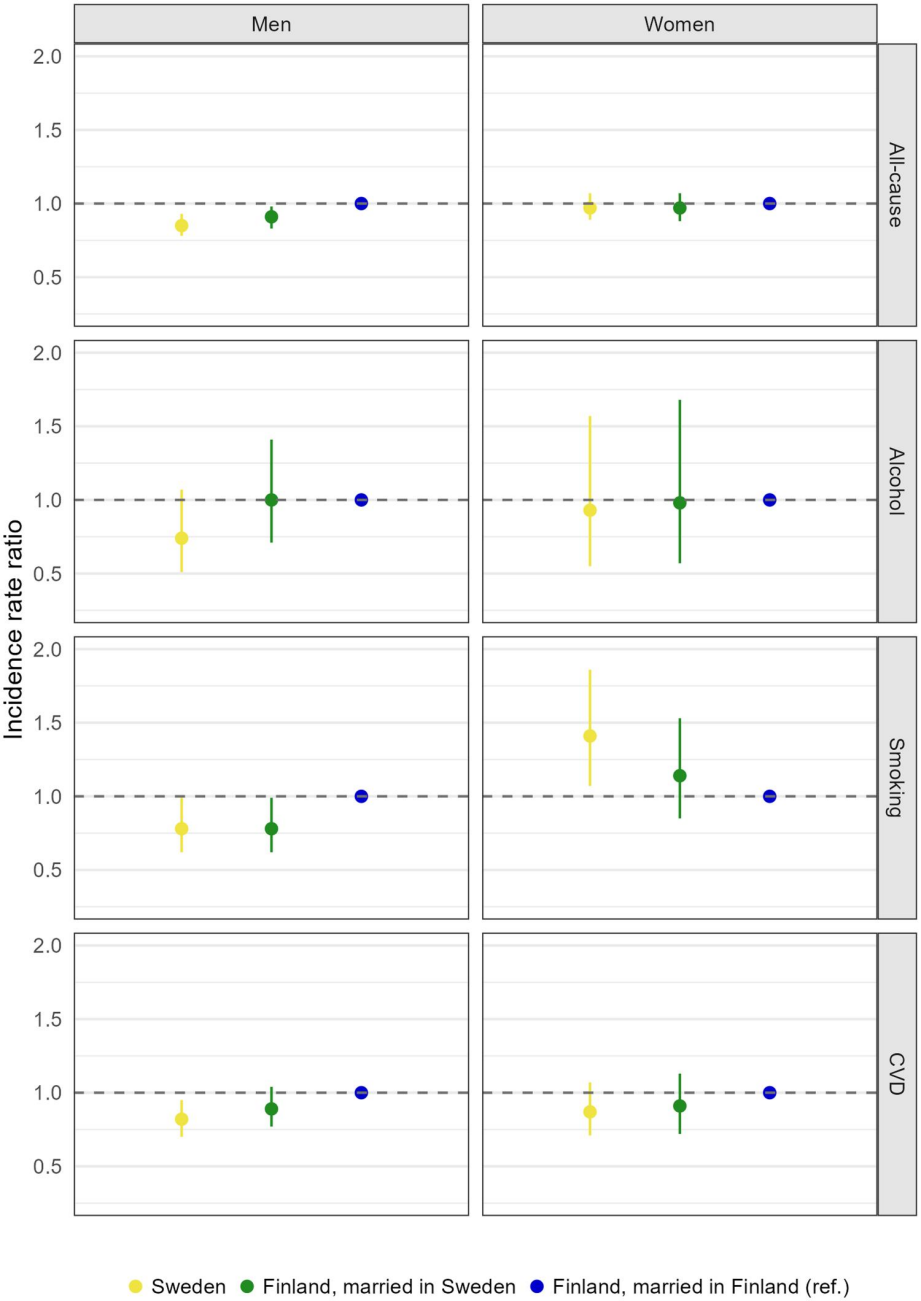
IRRs for all-cause and cause-specific mortality among Finnish migrants in Sweden by spouse’s country of birth and marriage status at arrival in 2000–17. IRRs adjusted for age, income, educational attainment, having children in Sweden and duration of marriage.

Table 2 displays results from individual-level regression analyses conducted on Finnish migrants, with additional adjustments for having children in Sweden and within the household, and the duration of marriage. Notably, following adjustment for the duration of marriage, the IRRs revealed lower alcohol-related mortality among migrant men married to Swedish-born spouses (IRR = 0.74, 95% CI 0.56–0.98; Model 3) compared to those with Finnish-born spouses. This association emerged only after accounting for the duration of marriage, as longer marital unions were associated with lower mortality. Marriages between two Finns were on average formed earlier than marriages between migrants and natives (Supplementary Table S1). Among women, adjusting for the duration of marriage resulted in a substantial attenuation in the excess risk of smoking-related mortality among intermarried individuals (IRR = 1.30, 95% CI 1.06–1.60). Furthermore, we observed a general tendency indicating that marriages between Finns formed after migration to Sweden exhibited mortality patterns more aligned with those observed among intermarried migrants, as opposed to marriages formed before the migrants moved to Sweden (Fig. 2).

**Table 2.** Incidence rate ratios (IRRs) for all-cause and cause-specific mortality by spouse’s country of birth, children in Sweden and duration of marriage among Finnish migrants in Sweden in 2000–2017. IRRs adjusted for age, income and educational attainment in all models

		Men			Women		
		Model 1	Model 2	Model 3	Model 1	Model 2	Model 3
		IRR (95% CI)	IRR (95% CI)	IRR (95% CI)	IRR (95% CI)	IRR (95% CI)	IRR (95% CI)
All-cause mortality							
Spouse’s country of birth	Finland (ref.)	1.00	1.00	1.00	1.00	1.00	1.00
	Sweden	0.93 (0.87–0.99)	0.93 (0.87–1.00)	0.91 (0.85–0.97)	1.01 (0.95–1.09)	1.01 (0.94–1.08)	0.99 (0.92–1.06)
Child in Sweden	No		1.22 (1.08–1.37)	1.16 (1.03–1.31)		1.31 (1.16–1.49)	1.27 (1.12–1.44)
	Yes (ref.)		1.00	1.00		1.00	1.00
	Yes, in household		0.89 (0.82–0.95)	0.86 (0.79–0.93)		0.82 (0.75–0.89)	0.81 (0.74–0.88)
Duration of marriage^a^	Short (ref.)			1.00			1.00
	Intermediate			0.85 (0.78–0.92)			0.87 (0.78–0.95)
	Long			0.83 (0.76–0.92)			0.87 (0.78–0.96)
Alcohol-related mortality							
Spouse’s country of birth	Finland (ref.)	1.00	1.00	1.00	1.00	1.00	1.00
	Sweden	0.84 (0.64–1.10)	0.85 (0.64–1.11)	0.74 (0.56–0.98)	1.02 (0.71–1.48)	0.99 (0.69–1.44)	0.94 (0.65–1.36)
Child in Sweden	No		1.39 (0.92–2.12)	1.04 (0.68–1.59)		2.57 (1.58–4.19)	2.28 (1.40–3.73)
	Yes (ref.)		1.00	1.00		1.00	1.00
	Yes, in household		0.77 (0.57–1.04)	0.61 (0.45–0.83)		0.71 (0.46–1.09)	0.64 (0.41–0.99)
Duration of marriage^a^	Short (ref.)			1.00			1.00
	Intermediate			0.56 (0.41–0.75)			0.92 (0.61–1.39)
	Long			0.38 (0.26–0.55)			0.63 (0.38–1.06)
Smoking-related mortality							
Spouse’s country of birth	Finland (ref.)	1.00	1.00	1.00	1.00	1.00	1.00
	Sweden	0.95 (0.79–1.15)	0.96 (0.79–1.16)	0.91 (0.75–1.10)	1.42 (1.17–1.72)	1.41 (1.16–1.71)	1.30 (1.06–1.60)
Child in Sweden	No		1.22 (0.89–1.67)	1.08 (0.78–1.50)		1.35 (0.96–1.89)	1.20 (0.85–1.69)
	Yes (ref.)		1.00	1.00		1.00	1.00
	Yes, in household		0.83 (0.67–1.01)	0.75 (0.60–0.94)		0.78 (0.62–0.99)	0.73 (0.57–0.93)
Duration of marriage^a^	Short (ref.)			1.00			1.00
	Intermediate			0.76 (0.60–0.97)			0.67 (0.52–0.87)
	Long			0.67 (0.52–0.87)			0.59 (0.45–0.78)
CVD mortality							
Spouse’s country of birth	Finland (ref.)	1.00	1.00	1.00	1.00	1.00	1.00
	Sweden	0.88 (0.77–1.00)	0.89 (0.78–1.01)	0.87 (0.77–0.99)	0.95 (0.81–1.11)	0.95 (0.80–1.11)	0.92 (0.78–1.09)
Child in Sweden	No		1.22 (0.99–1.51)	1.18 (0.95–1.46)		1.19 (0.89–1.59)	1.14 (0.85–1.54)
	Yes (ref.)		1.00	1.00		1.00	1.00
	Yes, in household		0.75 (0.65–0.86)	0.73 (0.63–0.84)		0.72 (0.59–0.89)	0.71 (0.58–0.88)
Duration of marriage^a^	Short (ref.)			1.00			1.00
	Intermediate			0.94 (0.80–1.11)			0.78 (0.61–0.98)
	Long			0.89 (0.74–1.05)			0.80 (0.64–1.01)

a Duration of marriage categorized in tertiles.

## Discussion

This study unveiled distinct mortality patterns among Finnish migrants in Sweden based on their spouse’s country of birth. Net of sociodemographic factors, Finnish migrant men married to Swedish-born spouses had lower rates of all-cause, alcohol-related, and CVD mortality compared to those married to Finnish-born spouses. Conversely, intermarriage was associated with higher smoking-related mortality among Finnish migrant women. The results indicate that intermarried migrants exhibited mortality patterns more akin to the native Swedish population, whereas those married to Finnish-born spouses demonstrated mortality rates closer to those observed among native Finns in Finland.

These findings contribute to our understanding of mortality convergence between migrants and natives over time [19, 23, 25], identifying intermarriage with a native-born spouse as a contributing factor. Previous studies on mortality have explored intermarriage’s association with very specific outcomes like COVID-19 and suicide [17, 18] and have not compared mortality patterns to those in the country of origin, thus being unable to assess mortality convergence. Our analysis delved into a broader spectrum of causes of death related to health risk behaviours, encompassing alcohol- and smoking-related as well as CVD mortality. Public health interventions should recognize the significant impact of both the migrants’ country of origin and their present circumstances in shaping health behaviours, ultimately influencing morbidity and mortality patterns.

Our results are consistent with the notion that marrying a native-born spouse can increase migrants’ exposure to local cultural norms and influence their health behaviours, including dietary choices, exercise routines, alcohol consumption, and smoking. When spouses are from the same origin, adaptation to the health behaviours of the destination country may be slower. We find that Finnish-born migrants married to Finnish-born spouses more closely share mortality patterns with the native Finnish population, especially if married before migrating to Sweden. This suggests that behavioural patterns established early in relationships may persist, and migrants already married before migration might be less inclined to adopt new habits in the destination country.

In addition to influences during marriage, migrants who marry natives may be more integrated into the local culture prior to marriage. They might have adopted the local habits and values and built extensive networks within the native community, which could increase their likelihood of marrying a native-born spouse. Such effects may be especially pertinent in the context of Finnish migration to Sweden, where migrants from Finland’s Swedish-speaking minority might have been likely to marry Swedish-born spouses due to the smaller language barrier. A study from Finland has shown that Swedish-speaking Finnish men have lower alcohol-related and CVD mortality, while Swedish-speaking Finnish women have higher smoking-related mortality than the Finnish-speaking majority [26]. Unfortunately, we were unable to identify the first language of migrants and therefore could not evaluate this possibility directly. Disentangling the causal and selection pathways would necessitate alternative datasets and study designs, offering a promising pathway for future research.

When there is convergence of behaviours, whether this positively or negatively impacts health is conditional on the migration context, specifically the patterns of health behaviours in the country of origin relative to the destination. For Finnish migrant men in Sweden, convergence implied an improvement in health behaviours and health status, manifested in lower all-cause, alcohol-related, and CVD mortality. Conversely, given the lower smoking-related mortality risk among women in Finland as opposed to Sweden (Fig. 1), convergence led to higher smoking-related mortality. This was also partially attributed to shorter durations of marriage among intermarried women compared to those married to Finnish-born spouses. Our findings are in line with a prior study that observed convergence in smoking rates towards the Swedish level among intermarried pregnant women with the direction of change depending on the smoking rates in the country of origin [27]. In contrast to men, we find no convergence in overall mortality among women, with both migrant groups having higher all-cause death rates compared to either of the native populations. This is consistent with previous findings showing higher all-cause mortality for Finnish migrant women in Sweden and likely reflects the sum of opposing trends in specific causes: increased smoking-related mortality but reduced alcohol and CVD mortality [19]. These patterns are more prominent among nonmarried women (Supplementary Fig. S2) and less pronounced among those who are married.

Overall, these findings suggest that marrying a native-born contributes to a convergence in health behaviours, aligning migrant mortality patterns with those of the population in the destination country. However, this observation does not negate the critical role played by structural determinants of mortality among migrants, notably socioeconomic position [28]. The comparison between adjusted IRRs in Fig. 1 and unadjusted IRRs in Supplementary Fig. S2 underscores that the higher socioeconomic position of intermarried migrants contributed to the observed mortality differentials based on the spouse’s country of birth.

It is important to note that Finnish migration to Sweden represents a unique historical context, and caution may be needed when generalizing the results to other—especially more culturally distant—migration groups. For instance, the dynamics of marital strain [29, 30] might play a more crucial role among spouses from culturally more diverse backgrounds, possibly leading to more substantial or qualitatively different consequences of intermarriage on health. While no single migrant group can represent all others, our analysis identifies intermarriage as a potentially important factor in mortality convergence. The magnitude of convergence, and whether it improves or worsens mortality, will nevertheless depend on the specific migration context.

### Methodological considerations

This study aimed to analyse migrant mortality patterns based on the spouse’s country of birth and compare these patterns to reference populations in both the country of origin and the destination. Access to population records from both countries was crucial for our research objectives, although sharing individual-level data across borders was not permitted [31]. We utilized a combination of direct matching and inverse probability weighting techniques to adjust for differences in the sociodemographic composition between the groups. This was complemented with analysis using individual-level register data, accounting for migration, marriage, and childbearing histories of individuals.

This study is subject to certain limitations. Our analysis centred on migrants married in 1999, thereby excluding individuals whose marriages had dissolved before this year. Additionally, we could not capture information regarding dating and cohabitation among nonmarried couples. People also select into marriage based on socioeconomic and health characteristics, and thus married people are more similar to each other than people picked at random. Further research more fully taking account of spouse’s characteristics could bring more understanding of the role of selection processes.

We censored migrants who returned to Finland during the follow-up period, which could influence mortality differences if return migration was associated with health selection. The number of migrants who emigrated from Sweden during the follow-up was small (*n* = 2557, 4.7%), and therefore unlikely influenced our main conclusions, though it should be noted that Finnish migrants married to a native Swede were more likely to remain in Sweden. Moreover, our inability to consider the health status at the time of migration and marriage formation further limits the comprehensive assessment of health convergence.

Age at migration is a crucial factor in understanding the integration process, as those who migrated before age 15 were most likely to marry a native Swede (Supplementary Fig. S3). This pattern suggests overlapping roles of age at arrival and intermarriage in the integration process. In our study, the cohort structure of the data meant that age at arrival was closely related to age during follow-up (Supplementary Fig. S4), preventing us from including both age variables in the same model. Future studies on populations with a greater cross-variation between age at arrival and age at follow-up using an open cohort design could better disentangle these intertwined processes.

## Conclusions

This study found that Finnish migrants in Sweden exhibit distinct mortality patterns based on their spouse’s country of birth. Among men, intermarriage with Swedish-born spouses was associated with lower all-cause, alcohol-related, and CVD mortality, while among women, it was linked to higher smoking-related mortality, aligning their mortality rates closer to those of native Swedes. These findings highlight how intermarriage influences mortality convergence between migrants and natives, with impacts varying by gender and cause of death.

## Supplementary Material

ckae179_Supplementary_Data

## Data Availability

The authors do not have permission to share data. Key points Finnish migrants in Sweden married to Swedish-born spouses showed mortality patterns more closely resembling those of the native Swedish population, while those married to Finnish-born spouses exhibited mortality rates similar to the Finnish population in Finland. Intermarried men displayed lower all-cause, alcohol-related, and CVD mortality rates, whereas intermarried women experienced higher smoking-related mortality rates compared to those married to Finnish-born spouses. Marrying a native-born individual might contribute to a convergence in health behaviours, aligning migrant mortality patterns more closely with those of the population in the destination country. Public health interventions should recognize the significant impact of both the migrants’ country of origin and their present circumstances in shaping health behaviours, ultimately influencing morbidity and mortality patterns.
