# Supplementary material for: Intermarriage and mortality among Finnish migrants in Sweden: a prospective register study using binational data
Source: Eur J Public Health. 2024 Nov 20;35(2):386–94. doi: 10.1093/eurpub/ckae179 (PMC11967888; doi:10.1093/eurpub/ckae179)

Intermarriage and mortality among Finnish migrants in Sweden: a prospective register study using binational data

Supplementary Material

Supplementary Table S1 The distribution of Finnish migrants in Sweden aged 40–64 years in 1999 by spouse's country of birth and country of marriage

	Men				Women			
	Finnish-born			Swedish-born	Finnish-born			Swedish-born
	All	Married in Finland	Married in Sweden		All	Married in Finland	Married in Sweden	
Income (%)								
Lowest quintile	8.0	9.3	7.3	7.1	8.7	10.5	7.4	6.9
2nd	18.5	20.1	17.4	15.5	20.0	22.2	18.5	15.1
3rd	27.9	27.8	27.9	24.9	28.0	27.7	28.2	21.9
4th	29.9	27.6	31.4	29.5	28.5	25.9	30.3	27.1
Highest quintile	15.7	15.1	16.1	23.1	14.8	13.7	15.6	29.1
Education (%)								
Compulsory	52.9	56.1	50.9	33.8	50.0	56.6	45.5	24.6
Intermediate	40.6	36.3	43.3	50.0	38.9	34.0	42.4	46.8
Tertiary	6.5	7.7	5.8	16.2	11.0	9.4	12.1	28.6
Child in Sweden (%)								
No	6.9	4.8	8.2	7.0	5.8	4.4	6.7	7.9
Yes	49.0	63.5	40.1	34.9	54.2	67.1	45.3	40.1
Yes, in household	44.0	31.6	51.7	58.1	40.0	28.6	48.0	52.0
Duration of marriage[a] (%)								
Short	25.9	5.2	38.7	50.0	32.9	3.5	34.8	42.9
Intermediate	37.5	31.3	41.3	29.1	35.8	28.3	41.1	34.5
Long	36.6	63.5	20.0	20.8	42.3	68.3	24.2	22.6

[a] Categorised in tertiles

Supplementary Figure S1 Age-adjusted incidence rate ratios (IRRs) for all-cause and cause-specific mortality among Finnish migrants in Sweden by spouse's country of birth as well as married native Swedes and Finns in 2000–2017.

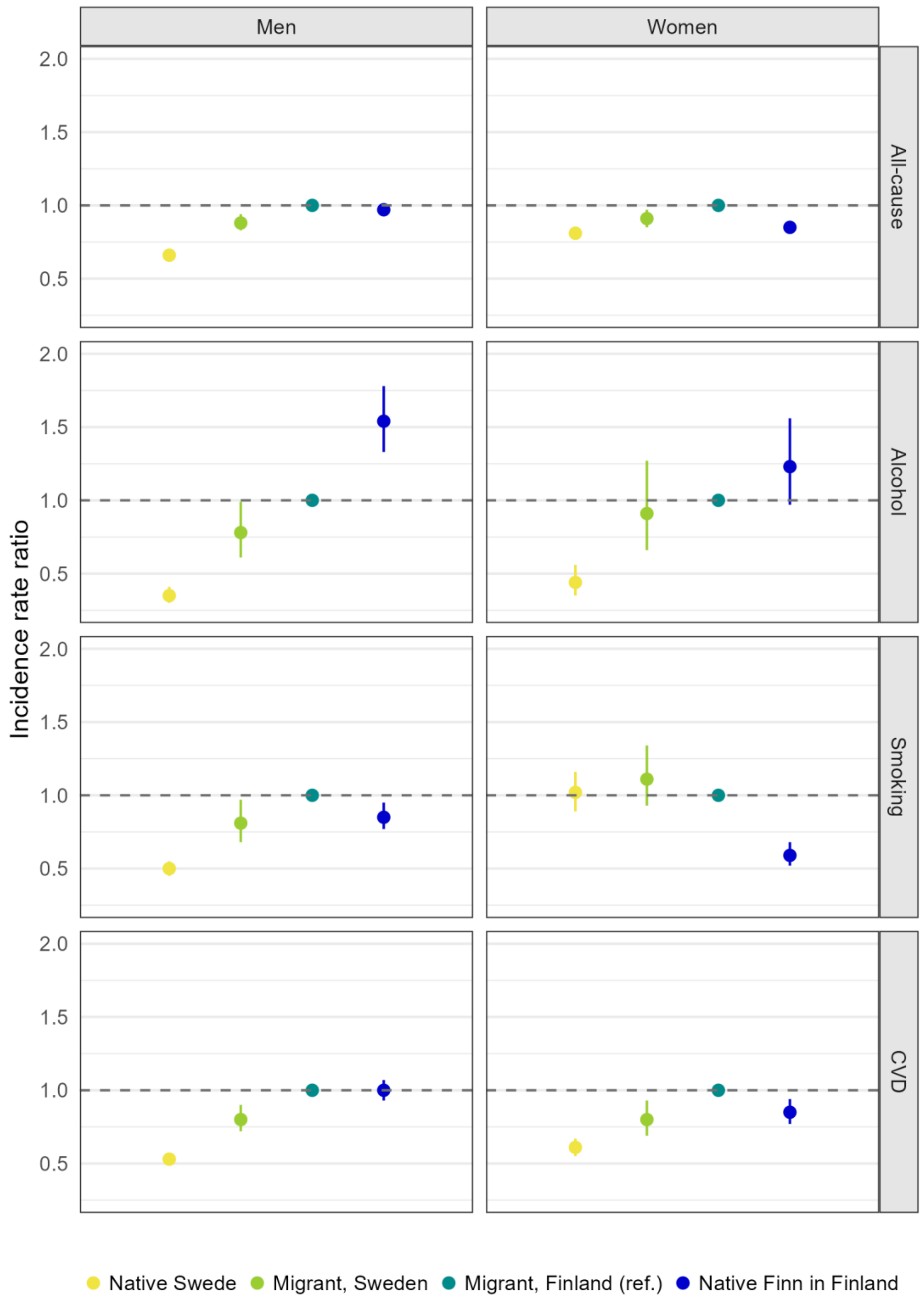

Supplementary Figure S2 Incidence rate ratios (IRRs) for all-cause and cause-specific mortality among non-married Finnish migrants in Sweden as well as non-married native Swedes and Finns in 2000–2017. IRRs adjusted by direct matching for age, income and educational attainment.

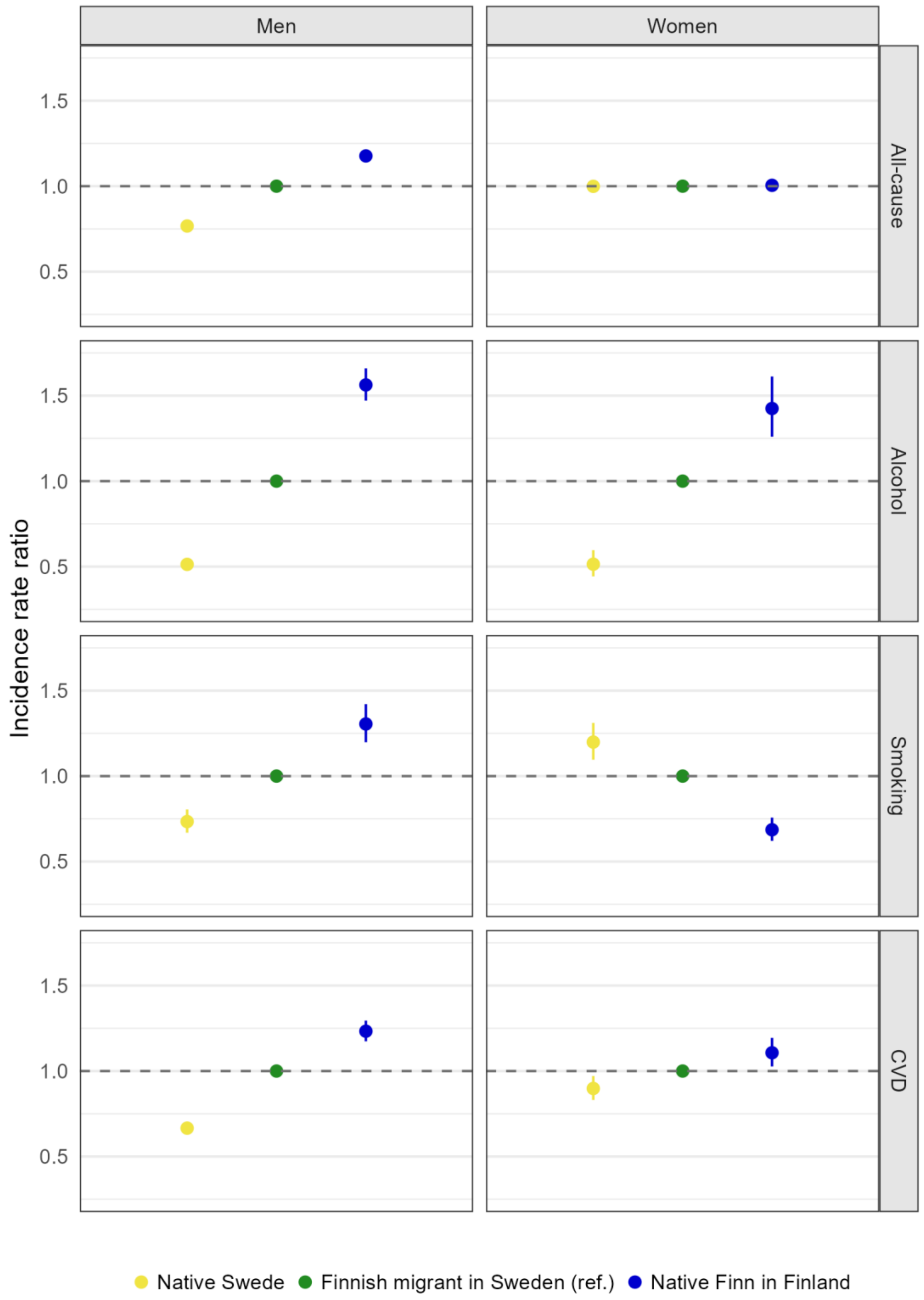

Supplementary Figure S3 Number of Finnish migrants in Sweden who are unmarried, married to Finnish-born spouses, and married to Swedish-born spouses in 1999, by age at arrival

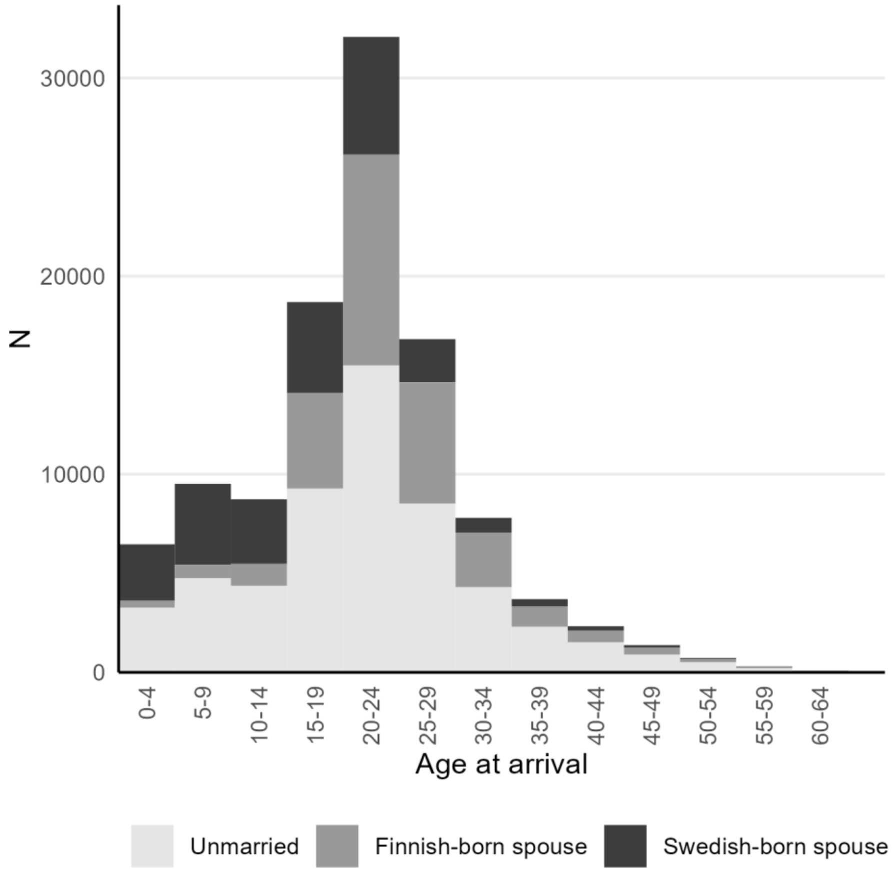

Supplementary Figure S4 Distribution of age at baseline by age at arrival among married Finnish migrants in Sweden in 1999

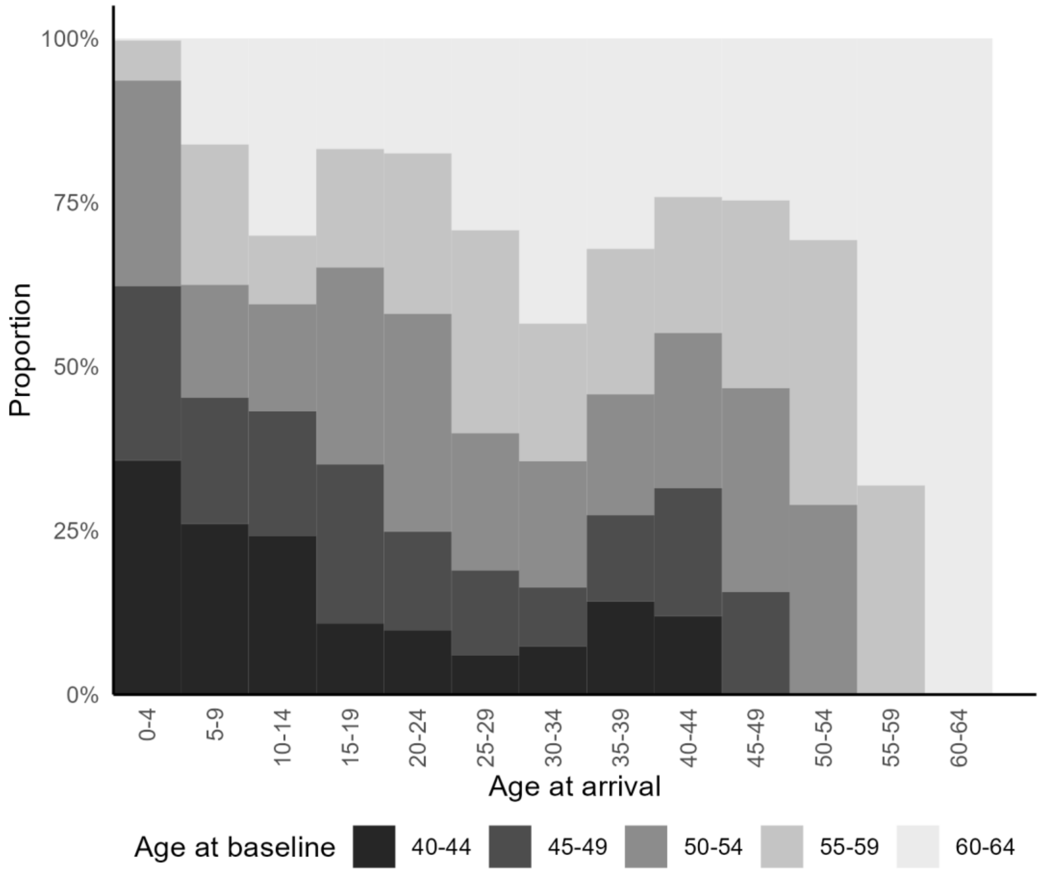

Supplement: ckae179_Supplementary_Data [file ckae179_supplementary_data.pdf]
